# Reply to Singh, G.D. Comment on “Dao et al. Retrospective Analysis of Real-World Data for the Treatment of Obstructive Sleep Apnea with Slow Maxillary Expansion Using a Unique Expansion Dental Appliance (DNA). *Pathophysiology* 2023, *30*, 199–208”

**DOI:** 10.3390/pathophysiology30040036

**Published:** 2023-10-08

**Authors:** Nhi Dao, Colette Cozean, Oleg Chernyshev, Clete Kushida, Jonathan Greenburg, Jonathan S. Alexander

**Affiliations:** 1 Department of Molecular and Cellular Physiology, Louisiana State University Health Shreveport, Shreveport, LA 71103, USA; hda001@lsuhs.edu; 2 EyeDeas Company, Lake Forest, CA 92630, USA; colettecozean@gmail.com; 3 Department of Neurology, Louisiana State University Health Shreveport, Shreveport, LA 71103, USA; oleg.chernyshev@lsuhs.edu; 4 Stanford Sleep Medicine Center, Redwood City, CA 94063, USA; clete@stanford.edu; 5 SnoreExperts, Los Angeles, CA 91602, USA; jgreenburg@outlook.com

In response to the commentary “Response to ‘Retrospective analysis of real-world data for the treatment of obstructive sleep apnea with slow maxillary expansion’” [1], the authors of the report would like to respond to several of the criticisms which were raised in the commentary.

The DNA device itself was cleared by the FDA under 510(k) for treatment of mild-to-moderate obstructive sleep apnea (OSA) based on the data included in this paper, instead of for palatal expansion only, a Class I indication.

One of our authors reviewed all papers for the introduction and body of the paper and did not include Dr. Singh’s articles because they did not meet our standards for inclusion (i.e., large sample size, controlled clinical trial or support of a key element of our discussion). We could have included several hundred papers in our footnotes, but limited the papers to those that supported or contradicted our discussion. Additionally, the study by Katz, et al. (2022) reported data on combinations of devices, some using mandibular advancement, others using expansion and others using a combination of the two. Although this article reviewed the same database, it had different inclusion/exclusion criteria and includes at least 3 appliances, some of which are mandibular advancement, some expansion, some both with 2 different connections between the trays for each. The current study excluded other modalities such as laser treatment, surgery, etc. and included safety data, which was not in the other study. Since these categories were not separated for statistical purposes, the authors could not compare to this paper.

As far as the authors know and as represented by the FDA, this is the first time, the FDA has cleared a palatal expander for OSA, making it available as a treatment of OSA to the general dentist and the public. 

There is not a general “current standard” for medical success according to the literature. The most frequently used standard for mandibular advancement oral therapy is a reduction in AHI of 50% or a resolution of OSA (AHI < 5). However, using either this standard or the standard applied by Dr. Singh, the data presented in this paper would have met the standard. We elected not to use either of these “standards” as the standard is for mandibular advancement, where AHI is usually measured with the appliance in the mouth and is not a “long-lasting” effect. During this study, AHI was measured without the appliance in the mouth and there are indications that the beneficial effects may be “long-lasting”. We do use another standard of 45% for resolution of AHI. How the DNA appliance works is described in detail in papers and publications by Dr. Singh. The authors do not believe there is yet sufficient evidence to support specific mechanisms described by Dr. Singh. Either way, the facts remain that the teeth did not ‘tip’ as this was evaluated in the protocol. 

As filed with and cleared by the FDA, the DNA appliance can consists of an upper tray, a lower tray or both. In this study, we used both. Dr. Singh may not be aware of this fact. We have not confused it with the mRNA. Similar data were taken with the mRNA. However, the mRNA has a connection between the two trays. The picture shown in the study is the DNA appliance. 

The author’s make no claim that the sample size for the additional modalities are statistically significant or that the correlations are statistically significant. However the authors, and one of the reviewers, felt this information was important to the reader as they may use the DNA appliance with other modalities in their practices.

Regarding ethnicity, the protocol collected data on ethnicities and all ethnicities enrolled in the study. There is no representation that the ethnicities matched the general ethnic demographic in the United States.

The statement we made that our findings are “comparable to the findings in all previous studies”, we believe to be fairly accurate. Our literature search did not reveal any articles from Dr. Singh that had a significant sample size to use in our comparison. 

We would agree that in order to properly state that “Since the 38 sites were distributed primarily across the United States, the data could be generalized to the US population” would require greater balance in the age and gender as well as more number of participants as a possible limitation of the current study.

In our description of the DNA device, the device is periodically adjusted to achieve expansion through screws which influence both the maxilla and mandibular width. The historical reference to the Fauchard appliance produces a slow maxillary expansion by buccal tipping. We are not aware of a contribution of ‘buccal tipping’ in our DNA approach. Buccal tipping was not listed as a safety concern reported at any of the 5 sites which provided data for the current study. The case report form requested information on whether buccal tipping was noted by providers and there were no reports of this complication. This distinction not only underlines the advancements in the design and functionality of the DNA device but also reinforces its safety and efficacy. As we move forward, we firmly stand by our approach and findings, and also remain open to that can shed more light on this topic.
